# Quick Process for Mercurial Ointment

**Published:** 1856-04

**Authors:** 


*Quick Process for Mercurial Ointment.*—M. Bernier, Pharmaceutist of Reuwez (Ardennes), recommends the following process. Take one-third of the lard to be used for the ointment, heat it in a skillet of copper till it commences to disengage vapors and burn, and then pour it into an earthen vessel, and place it in the cellar for ten or fifteen days. Use this lard to extinguish the mercury, employing an iron mortar, and observing to add the mercury gradually as each addition disappears. The mercury is soon perfectly extinguished when the rest of the lard is incorporated thoroughly, the whole operation requiring but an hour.—*Repertoire de Pharmacie and American Jour. of Pharmacy.*

